# Reply to “Geographical Accessibility Does Not Affect Prognosis After Living‐Donor Liver Transplantation”

**DOI:** 10.1002/ags3.70119

**Published:** 2025-10-30

**Authors:** Hajime Matsushima, Akihiko Soyama, Takanobu Hara, Ayaka Kinoshita, Takashi Hamada, Kazushige Migita, Ayaka Satoh, Hajime Imamura, Tomohiko Adachi, Susumu Eguchi

**Affiliations:** ^1^ Department of Surgery Nagasaki University Graduate School of Biomedical Sciences Nagasaki Japan

**Keywords:** accessibility, distance, living‐donor liver transplantation, outcome

We read with great interest the letter by Yugawa et al., sharing their experience at Kyushu University regarding the relationship between geographic distance and post‐transplant outcomes after living‐donor liver transplantation (LDLT) [1]. We appreciate their important contribution, which emphasizes that geographic distance alone does not affect post‐transplant outcomes when a specialized follow‐up system for distant patients is established.

The data from Kyushu University also highlight regional differences in the transportation infrastructure across Japan. In urban areas with extensive highways, railways, and airports, even patients residing a distance of > 40 miles can access tertiary care without delay. Conversely, in rural or geographically isolated regions, even shorter distances can impose serious barriers to the timely management of urgent complications because of the limited transportation options. Indeed, our prefecture includes remote islands with restricted transportation systems. This heterogeneity of accessibility may underlie the disparity between the findings of our study and theirs [1, 2]. In the United States, where cars are the predominant mode of transportation, distance is almost synonymous with accessibility, and outcomes have often been analyzed in terms of distance from transplant centers [3]. Meanwhile, in a country like Japan, where multiple transportation options exist and substantial regional differences are present, more detailed investigations that account for these regional disparities are warranted.

Although close collaboration with local hospitals and regular outreach visits by transplant specialists are effective strategies for optimizing long‐term outcomes after LDLT, additional measures are required in geographically isolated areas. Since the COVID‐19 pandemic, telemedicine has gained popularity for post‐transplant follow‐up [4, 5]. Remote follow‐up systems, including online consultations, laboratory monitoring, and digital collaborations with regional hepatologists, may provide a feasible pathway to secure the continuity of specialized post‐LDLT care, particularly for recipients in remote areas.

As LDLT expands throughout Japan, we believe that the long‐term management of recipients will increasingly require a nationwide, standardized follow‐up system that ensures equitable access to specialized care, irrespective of residence. This type of system should be supported by structured collaborations and networking among transplant centers, which would also foster the training of transplant hepatologists and surgeons across Japan. The establishment of such coordinated frameworks is essential to eliminate regional disparities and to further improve the long‐term outcomes after LDLT.

In conclusion, while geographic distance remains an important consideration, comprehensive nationwide strategies that integrate transportation accessibility, innovative technologies, and inter‐institutional collaborations are indispensable for overcoming geographic barriers and securing optimal long‐term outcomes for LDLT recipients.

## Author Contributions

Hajime Matsushima wrote the article. All authors participated in revising the article and approving the final draft.

## Conflicts of Interest

S.E. is an editorial board member of Annals of Gastroenterological Surgery.

## Linked Articles

This article is linked to https://doi.org/10.1002/ags3.70099.
